# Assessment of Malnutrition Among Adolescents:Can BMI be Replaced by MUAC

**DOI:** 10.4103/0970-0218.66892

**Published:** 2010-04

**Authors:** Aparajita Dasgupta, Arindam Butt, Tushar Kanti Saha, Gandhari Basu, Amitava Chattopadhyay, Anindya Mukherjee

**Affiliations:** Department of Community Medicine, A.I.I.H&.H, 110 C.R. Avenue, Kolkata-700 073, India

**Keywords:** Body mass index, middle upper arm circumference, sensitivity, specificity

## Abstract

**Objectives:**

To find out the magnitude of malnutrition among the adolescents of an urban slum of Kolkata study population. To compare the middle upper arm circumference (MUAC) with that of body mass index (BMI) for determination of nutritional status of the study population.

**Materials and Methods:**

This was a school-based descriptive epidemiological study done among adolescent male students aged 10–19 years in the service area of Urban Health Centre, Chetla. The school is an all boy’s government aided school and all the students reside in the Chetla slum, the largest slum of Kolkata. Anthropometric measurements of the students of one section selected from each class i.e. class V to XII were recorded.

**Results:**

Results showed 47.93% of study population as per BMI and 60.30% as per MUAC were malnourished. Evaluation of screening test showed MUAC as a marker was 94.6% sensitive and 71.2% specific. A correlation between measurements of MUAC and BMI was demonstrated (*r*=0.822; SE=0.035; 95% CI; P=0.000000; *r*^2^=0.74).

## Introduction

Malnutrition denotes impairment of health arising either from deficiency or excess or imbalance of nutrients in the body. Adolescence is an important period in the individual’s life. Adolescents represent around 20% of the global world’s population and around 84% of them are found in developing countries.(1) Adolescents constituted 22.8% of the population in India as on 1st March 2000.(2) Inadequate nutrition in adolescence can potentially retard growth and sexual maturation, although these are likely consequences of chronic malnutrition in infancy and childhood. Inadequate nutrition in adolescence can put them at high risk of chronic diseases particularly if combined with other adverse lifestyle behaviors the problem of malnutrition received recognition of planners and policy makers right from inception of five-year planning; a large number of national nutritional programs were implemented to combat the menace of malnutrition. However, malnutrition still persists. For individual assessment of body composition, anthropometry is being replaced by more accurate but also more complicated methods. Calculation of individual BMI from weight and height, however, still remains a valid tool for epidemiological studies on assessment of nutritional status especially at the community level. The aim of this study is to determine a tool that will be easier and more appropriate for screening of adolescent malnutrition. It was felt that assessment of nutritional status by middle upper arm circumference (MUAC) was easier, more convenient requiring less expertise than assessment with body mass index (BMI). The research question was while comparing BMI with MUAC can the latter may be considered as a better tool as far as accuracy and feasibility is concerned.

## Materials and Methods

Study area: In the service area of Urban Health Centre, Chetla, all high schools were registered first and from the list one school was selected as a study school by simple random sampling method. After taking prior permission from principal of the school, interview dates of study were fixed.

Study population: One section from each class (i.e. class V to XII) was selected by lottery method. All students aged between 10 to 19 years of selected sections were chosen as the study population. School record was used for getting reasonable accuracy in age assessment. Students who were present on the day of survey with above mentioned age were included in the study. Their MUAC measurement and value of BMI at different age points were compared with the corresponding reference value of National Health and Statistics report.(3)

Techniques and tools: All the students thus registered were subjected to anthropometric measurements. Middle upper arm circumference was measured in centimeter with a non-stretched measuring tape with the right arm hanging relaxed. The measurement was taken midway between the tip of the acromion and olecranon process. The tape was placed gently but firmly round the arm to avoid compression of soft tissue. Measurement was taken nearest to 0.1 cm. The weight was measured in kilogram without shoes using a standing weighing machine having precision of 0.5 kg. Checks on the scale were made routinely before recording the weight of each student and the pointer was adjusted to zero using the screw provided. The height was taken barefooted in centimeter using standard measuring tape. A vertical tape fixed perpendicular to the ground on the wall was used as the scale. This tape was non-stretchable. It was fixed with transparent adhesive tape and care was taken to see that there was no fold or tilting to any side. During the examination, also the scale was repeatedly checked for loosening of adhesive tapes or tilting of the scale. Height was recorded to the nearest 1 cm. The body mass index was calculated as weight in kg/height in m^2^.

Method of analysis: The data so collected were compiled in MS Excel and analyzed using Epi-info and Winpepi software. Two mean tests were carried out using Student’s ‘t’ test for comparing means of BMI and MUAC for normal and study population at each age point. A correlation between BMI and MUAC measurement was demonstrated.

## Results and Discussion

Proper food and good nutrition are essential for survival, physical growth, mental development, performance and productivity, health and wellbeing of adolescents. Almost half of the adolescents of both sexes are not getting even 70% of their daily requirements of energy and a quarter of them are getting less than 70% of RDA of proteins. Malnutrition is seen in 30% of adolescent girls and 18% boys.(4)

Therefore, assessment of all adolescents with the help of a simple, easy and at the same time accurate and quickly implemented method is the need of the hour. With extensive literature search, it was evident that most of the studies related to nutritional status were based on BMI. For BMI, patients were required to stand. Further measuring BMI requires a height board, weighing scales, and mathematical calculations. On the other hand, assessment by MUAC is easy, feasible and does not require a weighing machine carrying which sometimes becomes quite tiring especially when house to house survey is done. Again, MUAC does not require measuring the height which again sometimes is subject to inaccuracy as the floor may not be flat or there may not be enough light in the hovels or shanties that the surveyor visits during the survey at the community level. Moreover, measurement of MUAC can be performed on a client who is standing, sitting, or, in extreme cases even when lying down that is in an extremely sick person.

This study was a school-based descriptive epidemiological study of cross-sectional design done among 194 adolescent male students aged between 10 and 19 years. Majority (75.26%) of the adolescents were Hindus.

Table 1 shows mean BMI and MUAC of study population at different age points compared with the corresponding reference value of National Health and Statistics report. Result suggests the presence of under nutrition in almost all the age groups in respect to both BMI and MUAC.

**Table 1:** BMI (mean ± SD) and MUAC (mean ± SD) of study population according to age(*n*=194)

Age in years	Number (%)	Observed BMI (kg/m^2^)	**Ref. value BMI (kg/m^2^)	Difference of mean BMI	Observed MUAC (cm)	**Ref. value MUAC(cm)	Difference of mean MUAC
		Mean ± SD			Mean ± SD		
10	4(2.06)	15.94 ± 2.03	19.6 ± 4.31	3.66(NS)*	19.5 ± 1.77	23.4 ± 4.04	3.9(NS)
11	7(3.60)	15.44 ± 2.10	20.5 ± 6.78	5.06(NS)	18.11 ± 1.96	24.6 ± 6.82	6.49(S)
12	20(10.30	15.44 ± 1.90	21.0 ± 7.29	5.56(S)*	18.5 ± 2.18	25.4 ± 6.91	6.9(S)
13	41(21.13)	17.58 ± 3.86	21.8 ± 6.74	4.22(S)	21.11 ± 3.41	26.8 ± 6.38	5.69(S)
14	41(21.13)	17.73 ± 3.05	22.0 ± 8.22	4.27(S)	21.64 ± 3.19	27.7 ± 6.41	6.06(S)
15	22(11.34)	17.13 ± 2.54	23.2 ± 5.91	6.07(S)	21.29 ± 3.14	29.3 ± 5.39	8.01(S)
16	35(18.04)	19.58 ± 3.61	24.5 ± 7.5	4.92(S)	23.37 ± 2.61	30.8 ± 6.42	7.43(S)
17	16(8.24)	20.13 ± 3.69	24.0 ± 7.50	3.87(S)	24.28 ± 2.23	30.7 ± 5.94	6.42(S)
18	7(3.06)	18.21 ± 1.56	24.6 ± 9.08	6.39(NS)	22.78 ± 1.75	31.5 ± 7.0	8.72(S)
19	1 (0.51)	24.36 ± 0.00	25.6 ± 8.72	1.24	27.50 ± 0.00	32.4 ± 6.68	4.9
Total	194(100)	−	−		−	−

* Significance test: t test, NS: not significant, S: Significant (P<0.05)

** Reference mean of BMI, MAUC adopted from NHANES (National Health and Nutritional examination Survey) 2003-06 conducted by Centres for Disease Control (CDC), National Centre for Health Statistics (NCHS).

It is observed in Table 2 that the proportion of under nourishment was 47.93% and 60.30% according to BMI and MUAC, respectively.

**Table 2:** Frequency distribution of adolescents in relation to their nutritional status according to their BMI and MUAC (*n*=194)

Nutritional status	BMI	(kg/m^2^) MUAC (cm)
5^th^ to 95^th^ Percentile (normal)	101 (52.06)***	77 (39.69)
<5^th^ Percentile (undernourished)	93 (47.93)	117 (60.30)
Total	194 (100)	194 (100)

*** Figures in parentheses indicate percentage.

Results of screening test [Table 3] and evaluation [Table 4] using MUAC show the test to be 94.6% sensitive and 71.2% specific.

**Table 3:** Screening test result for assessment of nutritional status with MUAC among the respondents (*n*=194)

Malnutrition(MUAC)	Malnutrition(BMI)		Total
	Yes	No
Yes 88	(TP)	29 (FP)	117 (TP+FP)
No	5 (FN)	72 (TN)	77 (FN+TN)
Total	93 (TP+FN)	101 (FP+TN)	194 (TP+FP+FN+TN)****

**** TP - True positive; FP - False positive; FN - False negative; TN - True negative.

**Table 4:** Evaluation of screening of nutritional status by MUAC

Measures	Results
Sensitivity	94.6%
Specificity	71.2%
Predictive value of positive test	75.2%
Predictive value of negative test	93.5%
Percentage of false negative	5.37%
Percentage of false positive	28.7%
Positive likelihood ratio	3.284
Negative likelihood ratio	0.075

**Figure 1:**
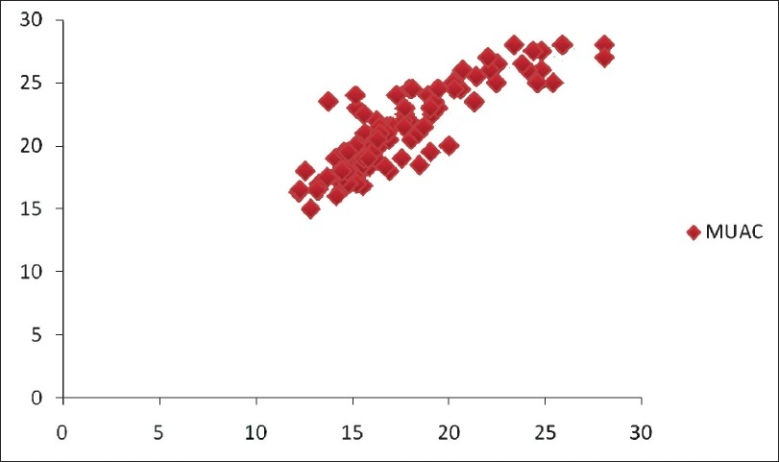
Scatter diagram showing linear relationship between BMI and MUAC

Figure 1 shows a strong correlation between BMI and MUAC. A significant correlation between measurements of MUAC and BMI was demonstrated (*r*=0.822; SE=0.035; 95% CI; P=0.000000; *r*^2^ =0.74).

A study on adolescent nutrition in a rural community in Bangladesh by A.K.M. Shahabuddin *et al* shows 67% of adolescents were thin.(5) Another study by K Anand *et al* on “nutritional status of adolescent school children in rural North India” shows thinness was present in 43.8% of the boys and 30.1% of the girls.(6) A study by A.N. Kanade *et al* on “under nutrition and adolescent growth among rural Indian boys” shows mean BMI values for broad age groups, viz., <12 years, 12–14 years, 14–16 years and 16–18) years were 13.81, 14.35, 15.43 and 16.63,respectively. More than 70% of the boys had in fact BMI values as low as IS, indicating Frank under nutrition.(7) A study of nutritional status of adolescent girls in the rural areas of Varanasi by Seema Choudhary, C.P. Mishra and K.P. Shukla among 270 adolescent girls shows 68.52% were undernourished (BMI<18.5), and average mid arm circumference was 82.81% of corresponding reference value.(8) A study on comparison between body mass index, triceps skin fold thickness and mid arm muscle circumference in Saudi adolescents by Bahaa Abalkhail, Sherine Shawky shows the P85 and P95 for BMI were higher for Saudi adolescents than the NHANES I. Conversely, there was a lower mid arm muscle circumference at P90 and P95 than the NHANES I reference population curves.(9) Using middle upper arm circumference to assess severe adult malnutrition during famine, a study by S. Collins shows the proportions of the population and the actual individuals identified as malnourished by the 2 indicators were similar. A correlation between measurements of MUAC and BMI demonstrated, *r*=0.88; 95% confidence interval, 0.82–0.92 *P*<.001.(10)

All the above mentioned studies showing a high magnitude of under nutrition throughout the country and results were more or less similar to our study.

Many studies have reported that gross weakness and flexor contractions prevented measurements of weight or height in a substantial proportion of severely undernourished adults. Moreover, the necessary equipment, including scales and height boards, may not be available. Difficulties in the calculation of the indices – the calculation of BMI and Rohrer Index – may be unfamiliar to field workers and therefore more difficult to use than other anthropometric indices. Indices comparing weight and height cannot be used to assess pregnant adolescents. Because of the extra weight of the fetus, other products of conception, and added maternal tissue, indices using weight and height may not accurately indicate the nutritional status of pregnant adolescents. During pregnancy, other measures, such as weight gain during pregnancy or MUAC, must be used to judge nutritional status.

## Conclusion

In this study, it can be stated that the mid arm circumference measurement is a reliable and a feasible method of assessment of nutritional status of adolescents. However, the researchers feel that there are some aspects of MUAC that should be kept in mind. MUAC changes substantially with age during adolescence. As a result, a different cut-off point must be used for adolescents of different ages. This requires an accurate age for each survey subject in order to judge whether they fall above or below an age-specific cut-off point. Again in spite of the convenience and ease of measurement of MUAC, it requires careful training and supervision like in order to prevent wrapping the measuring tape too tightly or too loosely, which results in an erroneous estimate and some degree of observer variability. It is felt that this is a very humble effort to identify a simple method of nutritional assessment of adolescents and therefore it is suggested that more research is done on newer methods of nutritional assessment in a larger sample and in many countries so that these methods are adapted by all, especially at the community level.
